# In vitro regeneration approaches for restoration of *Ceropegia mohanramii—*an endemic and critically endangered asclepiad

**DOI:** 10.1186/s43141-019-0003-6

**Published:** 2019-09-23

**Authors:** Avinash A. Adsul, Jaykumar J. Chavan, Nikhil B. Gaikwad, Rajaram V. Gurav, Ghansham B. Dixit, Shrirang R. Yadav

**Affiliations:** 1 Department of Botany, Gokhale Education Society’s Arts, Commerce and Science College, Jawhar, 401603 India; 2 Department of Botany and Biotechnology, Yashavantrao Chavan Institute of Science, Satara, 415001 India; 3 Department of Botany, Shivaji University, Kolhapur, 416004 India

**Keywords:** *Ceropegia mohanramii*, Conservation, Endemic, Endangered, Micropropagation, Ornamental, Restoration

## Abstract

The study aimed to develop an efficient, rapid, and large-scale in vitro regeneration system for propagation, conservation, and restoration of an endemic and critically endangered herb, *Ceropegia mohanramii*. The cultures were established using nodal explants on Murashige and Skoog’s (MS) medium supplemented with 6-benzylaminopurine (BAP: 1.0 mg/l). Nodal buds cultured on MS medium supplemented with BAP (2.0 mg/l) along with indole-3-butyric acid (IBA, 0.5 mg/l) resulted with production of maximum number of shoots (17.1 ± 1.2) in hundred percent of the cultures. MS medium supplemented with BAP (2.0 mg/l) along with diverse concentrations of indole-3acetic acid (IAA) promoted the in vitro flowering. In vitro regenerated shoots were transferred to one-half MS medium fortified with singular supplementation of auxins, where IBA (1.5 mg/l) served optimal for production of maximum number of roots (5.7 ± 0.6). In vitro derived plantlets were hardened under controlled conditions in a glasshouse and subsequently transferred to soil. Over 1200 saplings were transplanted to eight different localities of the Western Ghats where over 76% survival is recorded after 1 year of transplantation.

## Introduction

The genus *Ceropegia* (family—Apocynaceae) is a group of more than 220 herbs and climbers which displayed wide diversity among habit, habitat, flower architecture, and ecological adaptations [1, 2]. Out of 57 Indian *Ceropegia* spp., 40 have been reported alone from the Western Ghats of which 35 are endemic to the region [3]. Most of the species are known for endemism and their existence is restricted to remote pockets in two mega diversity hotspots viz. the Western Ghats and the Himalaya. The group is very interesting from the diversity, rarity, phytogeography, and pollination biology point of view [2]. Moreover, many species have nutritional, pharmacological, ornamental, and medicinal implications [4, 5].

Currently, *Ceropegia* spp. from wild has been exploited continuously leading to depletion of natural populations and genus as a whole is under threat. *Ceropegia mohanramii* Yadav, Gavade and Sardesai is one of the recently described endemic species occurring naturally in the Northern Western Ghats of India [6]. It is an erect, perennial, and tuberous herb that grows on low altitude lateritic plateaus [2]. Meticulous survey indicated the non-occurrence of this species in its probable area of occurrence, and now, this species is considered as critically endangered. Moreover, only a few individuals are remaining in its type locality. As this species grows on plateaus, grazing by herbivore feeds along with grasses is the major hurdle for its survival in the nature [2]. So there is an urgent need to check the depletion of the natural population at the earliest by the implication of the latest available biotechnological tools. Plant tissue culture is one of the viable routes for large-scale propagation, conservation, and restoration of threatened plants [5, 7]. In the present study, in vitro regeneration system including shoot multiplication, rooting, and hardening has been developed for *C. mohanramii*. Moreover, the hardened and acclimatized plantlets were transplanted to natural localities in the Western Ghats of India.

## Material and methods

Young and healthy shoots were collected from the naturally grown plantlets of *C*. *mohanramii* at Kochara in Northern Western Ghats of India. Leaves were removed and shoots were cut into segments and thoroughly washed under running tap water. Then, the shoots were transferred to detergent (Labogent 0.5% v/v) for 15 min. The shoots were surface sterilized in freshly prepared aqueous HgCl_2_ solution (0.1%) for 5 min and transferred to sterile distilled water (three times each for 5 min). After thorough washing, the stem segments were cut into single node explants and transferred to MS medium [8] supplemented with various concentrations and mixtures of cytokinins and auxins (BAP, TDZ, IBA, and IAA). The culture tubes were kept in the incubation room supplied with light (35 μmol m^−2^ s^−1^) and controlled temperature (25 ± 1 °C). Microshoots with 2–3 pairs of leaves from optimal media composition were transferred to half-strength MS medium fortified with auxins (IAB and IAA) for in vitro rooting.

Plantlets with well-developed root and shoot system were removed from the culture vessels and washed under running tap water for removal of traces of culture media. Then, the roots were treated with fungicide Bavistin (0.5% w/v) for 5 min to avoid the contamination, and the shoots were washed in sterile water to remove the traces of fungicide. The plantlets were transferred to sterile planting substrate (a mixture of sand and coco peat 1:1) for hardening. Initially, the plantlets were covered and kept in the laboratory conditions for 2 weeks. The watering was done with sugar-free liquid MS medium where cent percent plants survived (1235 in vitro derived clones). The plantlets were then transferred to pots filled with soil were kept in glasshouse conditions (light, 45 μmol m^−2^ s^−1^; temp, 25 ± 1 °C; and humidity, 70%) for large-scale hardening (1235 plantlets). The well-acclimatized plantlets (1 month in glasshouse conditions) were transferred and reintroduced at different localities in the Western Ghats of India. The experiments were carried out for three times with 20 replicates, data were analyzed by one-way ANOVA, and the values were compared by Dunnett multiple comparison test (GraphPad Software, Inc, USA).

## Results and discussion

**Fig. 1:**
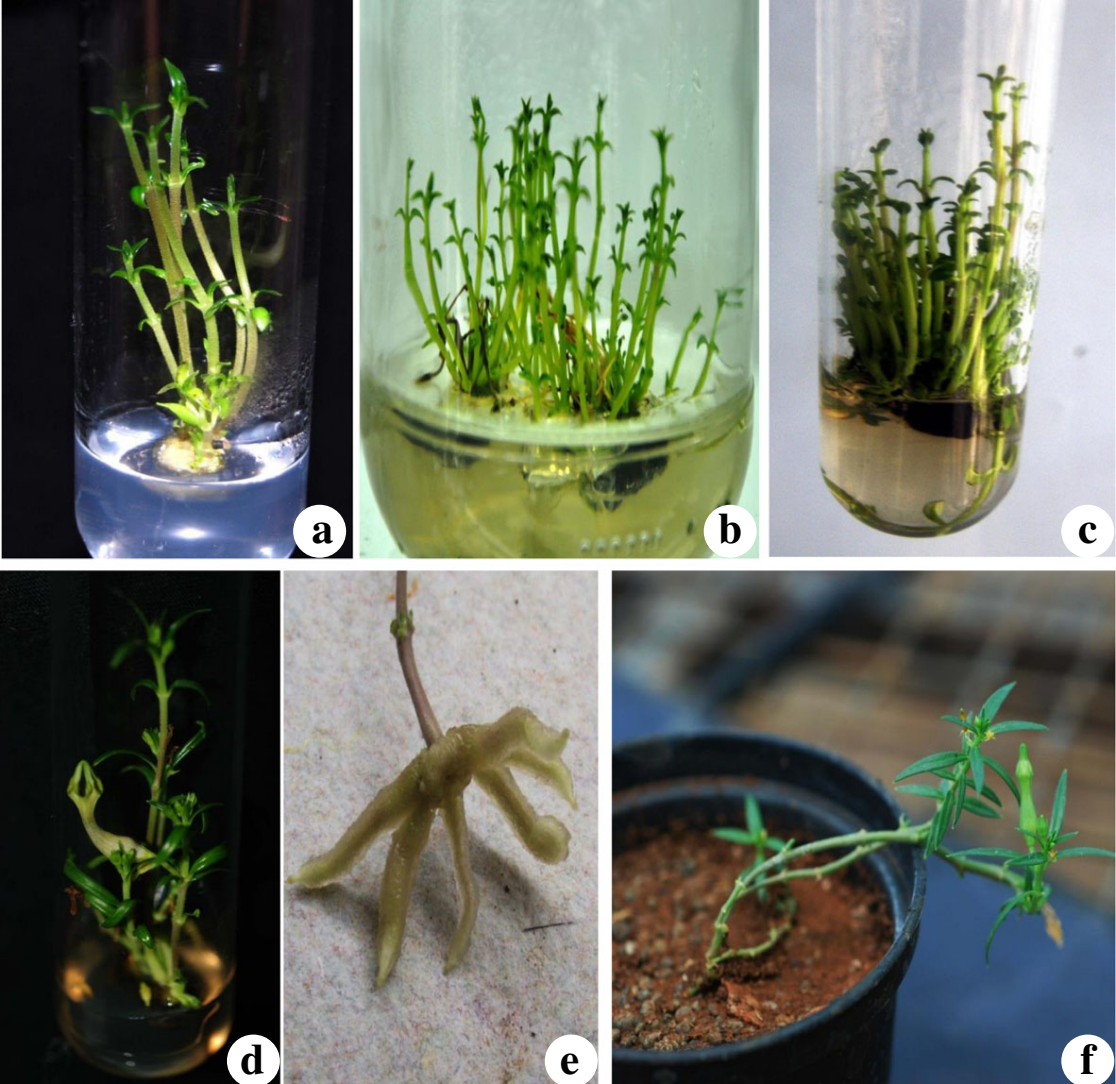
Micropropagation of *C. mohanramii*. **a** Shoot initiation (MS + BAP 1.0 mg/l). **b**, **c** Shoot multiplication (MS + BAP 2.0 + IBA 0.5 mg/l). **d** In vitro flowering (MS + BAP 2.0 + IAA 1.5 mg/l). **e** In vitro rooting (½ MS + IBA 1.5 mg/l). **f** Hardened plant

Nodal explants cultured on plant growth regulator (PGR)-free MS medium failed to induce the shoots. However, incorporation of singular or combinations of cytokinins and auxins (BAP, TDZ, IBA, and IAA) in the culture medium promoted the shoot induction and proliferation (Table 1). BAP was found most effective for the production of shoots as compared to TDZ. BAP alone at 2.0 mg/l produced 6.1 ± 0.3 shoots in 90% of the culture vessels (Fig. 1a). Similarly, efficacy of BAP towards production of multiple shoots was reported for *C. candelabrum* [9] and *C. noorjahaniae* [10]. TDZ (2.0 mg/l) produced 3.2 ± 0.2 numbers of shoots, and it was observed that the increased concentrations resulted in less number of shoots as well as stunted growth. However, Chavan et al. [11] reported the efficiency of TDZ in combination with BAP on shoot multiplication in *C. spiralis*. In the present study, a combination of BAP (2.0 mg/l) and IBA (0.5 mg/l) resulted in the production of the highest numbers of shoots (17.1 ± 1.2) in cent percent of the culture vessels (Table 1, Fig. 1b, c). MS medium supplemented with BAP (2.0 mg/l) along with IAA (1.0 mg/l) produced significant numbers of shoots. A combined effect of cytokinins and auxins has been reported in *C. panchganiensis* [12] and *C. bulbosa var. lushii* [13]. Now a day, new classes of plant growth regulators including meta-topolin are routinely used for in vitro propagation of orchids [14]. Such new class of growth regulator might be useful in future for in vitro propagation studies of threatened *Ceropegia* spp. In the present study, subsequent subcultures on same media composition also enhanced the shoot multiplication rate. Interestingly, increased concentrations of IAA along with BAP (2.0 mg/l) promoted the in vitro flowering (Fig. 1d). These observations are in line with previous reports on in vitro flowering of *Ceropegia* spp. [15].

**Table 1:** Efficiency of different cytokinins and auxins on shoot proliferation and in vitro rooting of *C. mohanramii*

Sr. no.	Plant growth regulator (mg/l)				Response (%)	No. of shoots ± SE	No. of roots ± SE
	BAP	TDZ	IBA	IAA			
1	PGR free				00	00	–
2	1.0	–	–	–	65	1.3 ± 0.3^ns^	–
3	2.0	–	–	–	90	6.1 ± 0.3**	–
4	3.0	–	–	–	90	4.4 ± 0.6**	–
5	4.0	–	–	–	75	4.0 ± 0.6**	–
6	–	1.0	–	–	60	1.5 ± 02^ns^	–
7	–	2.0	–	–	75	3.2 ± 0.5*	–
8	–	3.0	–	–	70	2.0 ± 0.4*	–
9	–	4.0	–	–	65	1.3 ± 0.4^ns^	–
10	–	–	0.5	–	85	–	2.0 ± 0.4*
11	–	–	1.0	–	85	–	2.2 ± 0.4*
12	–	–	1.5	–	95	–	5.7 ± 0.6**
13	–	–	2.0	–	80	–	3.8 ± 0.3**
14	–	–	–	0.5	75	–	3.5 ± 0.2**
15	–	–	–	1.0	65	–	3.0 ± 0.6*
16	–	–	–	1.5	60	–	2.1 ± 0.8*
17	–	–	–	2.0	60	–	2.0 ± 0.2*
18	2.0	–	0.5	–	100	17.1 ± 1.2**	–
19	2.0	–	1.0	–	95	10.2 ± 0.6**	–
20	2.0	–	1.5	–	95	10.2 ± 0.3**	–
21	2.0	–	2.0	–	80	4.2 ± 0.6**	–
22	2.0	–	–	0.5	75	4.5 ± 0.2**	–
23	2.0	–	–	1.0	80	5.3 ± 0.5**	–
24	2.0	–	–	1.5	85	4.1 ± 0.9**	–
25	2.0	–	–	2.0	80	4.0 ± 0.5**	–

Mean ± S.E. of 20 replicates per treatment. Values are significantly different when compared by Dunnett multiple comparison test (*ns* non-significant, *P < 0.05 and **P < 0.01)

Microshoots with well-developed leaves were transferred to half strength MS medium supplemented with auxins (IBA and IAA) for in vitro rooting (Table 1). PGR-free MS medium failed to induce the roots. MS medium supplemented with IBA (1.5 mg/l) served optimal for induction of maximum numbers of roots (5.7 ± 0.6) (Table 1, Fig. 1e). Rooting of the microshoots of most of the *Ceropegia* spp. was achieved through the incorporation of auxins especially IBA in the culture media [5]. Lower concentration of IAA (0.5 mg/l) also produced significant numbers of roots; however, the enhanced concentrations resulted in production of callus at the cut ends of microshoots. Plantlets with well-developed shoots and roots were transferred to sterile substrate (a combination of coco peat and sand) for hardening in the laboratory and subsequently transferred to soil (Fig. 1f).

**Fig. 2:**
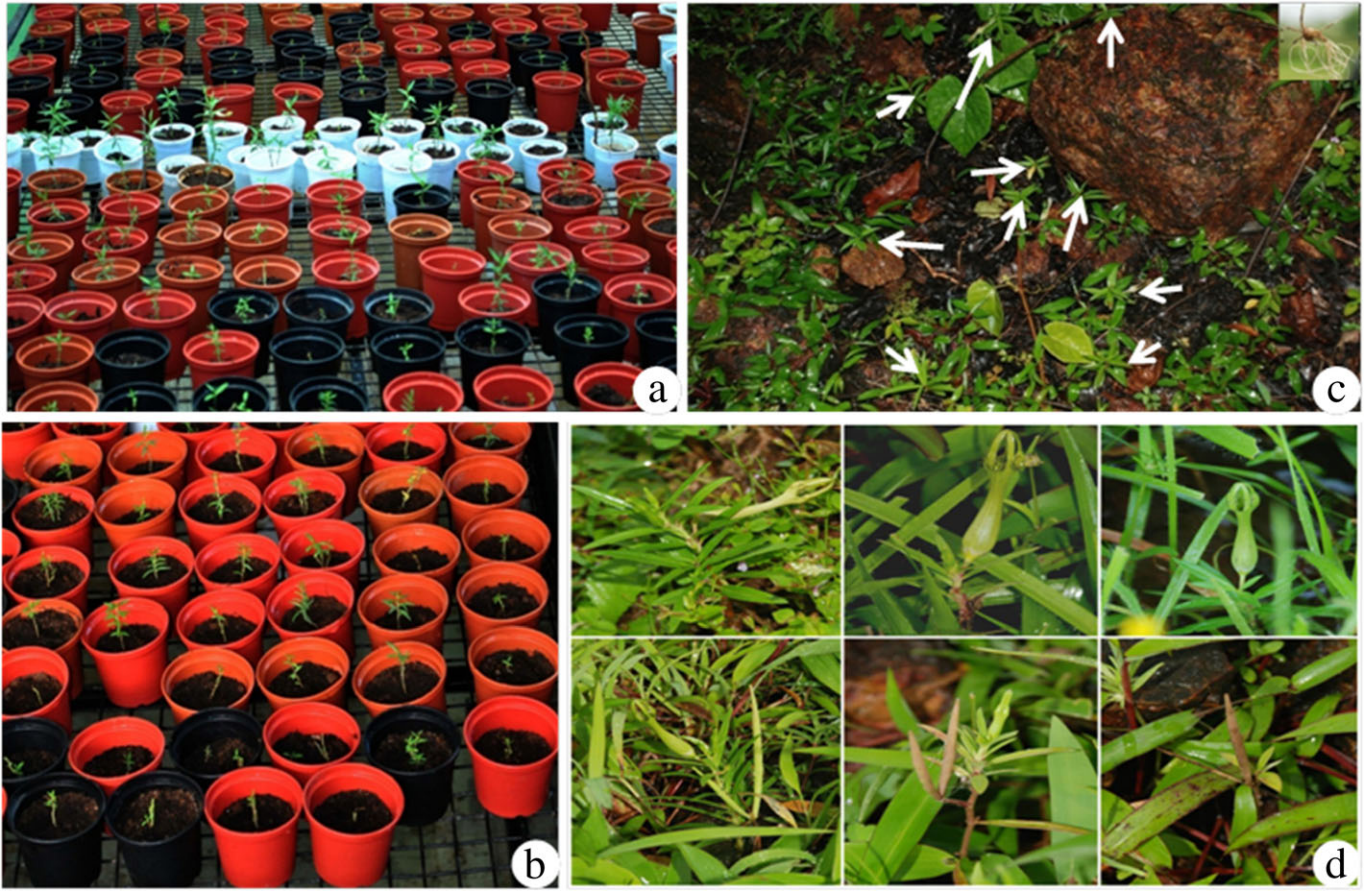
Reintroduction of micropropagated plantlets into natural localities. **a**, **b** Large-scale hardening. **c** Reintroduced plantlets. **d** Regeneration in natural habitat

The transplantation of in vitro raised plantlets into natural habitats is viable approach for plant conservation and restoration [5, 16]. In the current investigation, in vitro raised plantlets were kept in the glasshouse conditions for large-scale hardening (Fig. 2a, b). Over 1200 micropropagated samplings were transplanted to eight different natural localities in the Western Ghats of India (Table 2, Fig. 2c). After a year, the plants showed self-perpetuation at new site and exhibited over 76% of the establishment in natural conditions. The newly established populations showed health growth of plantlets under field conditions (Fig. 2d). Incessant monitoring on developmental progression confirmed their successful restoration. Similarly, the reintroduction of plantlets into natural habitats has been successfully demonstrated for *C. fantastica* [17] and *Hubbardia heptaneuron* [18].

**Table 2:** Reintroduction profile of micropropagated plantlets of *C. mohanramii* at different localities of the Western Ghats

Sr. no.	Name of the locality	GPS readings	No. of saplings transferred	No. of saplings survived	Survival rate (%)
1.	Gijkada, (Radhanagari WLS)	N 16, 19.304′ E 73, 52.786′	160	113	70.6
2.	Guhagar	N 17, 13.663′ E 73, 24.823′	130	75	57.6
3.	Pochari	N 17, 09.570′ E 73, 27.045′	80	69	86.2
4.	Ratnagiri	N 16, 56.122′ E 73, 18.203′	80	61	76.2
5.	Pavas	N 16, 52.826′ E 73, 19.649′	140	121	86.4
6.	Ganpatipule I	N 17, 07.135′ E 73, 16.602′	145	100	68.9
7.	Ganpatipule II	N 17, 07.34′ E 73, 16′ 31.02″	268	194	72.3
8.	Shiroda	N 15, 55′ 52.02″ E 75, 43′ 25.32″	210	187	89.0
Total			1213	920	76.6

## Conclusion

An efficient and rapid micropropagation protocol has been established for large-scale production of *C. mohanramii* for first time. The developed protocol resulted in the conservation and restoration of wild populations of *C. mohanramii* through replenishment by introducing in vitro derived plantlets into natural habitats. This protocol will be helpful for propagation and conservation of rare, endangered, and threatened plants.

## Data Availability

Not applicable

## Abbreviations

BAP: 6-Benzylaminopurine
IAA: Indole-3acetic-acid
IBA: Indole-3-butyric acid
MS: Murashige and Skoogs (1962)
PGR: Plant growth regulator
TDZ: Thidiazuron

## References

[1] Yadav SR (1996). Flytrap flowers of Western Ghats. Hornbill.

[2] Kambale SS (2015). Taxonomic revision of genus *Ceropegia* L. in India.

[3] Karthikeyan S, Sanjappa M, Moorthy S (2009) Flowering plants of India, Dicotyledons. Vol. I, (Acanthaceae-Avicenniaceae). pp. 160–164

[4] Chavan JJ, Gaikwad NB, Kshirsagar PR, Dixit GB (2013). Total phenolics, flavonoids and antioxidant properties of three *Ceropegia* species from Western Ghats of India. South Afr J Bot.

[5] Chavan JJ, Gaikwad NB, Dixit GB, Yadav SR, Bapat VA (2018). Biotechnological interventions for propagation, conservation and improvement of ‘Lantern Flowers’ (*Ceropegia* spp.). South Afr J Bot.

[6] Yadav SR, Gavade MN, Sardesai MM (2006) A new species of *Ceropegia* L. (Asclepiadaceae) from Konkan, Maharashtra, India. Rheedea 16(1):33-36, 2006

[7] Bapat VA, Yadav SR, Dixit GB (2008). Rescue of endangered plants through biotechnological applications. Nat Acad Sci Let.

[8] Murashige T, Skoog F (1962). A revised medium for rapid growth and bioassays with tobacco tissue cultures. Physiol Plantarum.

[9] Beena MR, Martin KP, Kirti PB, Hariharan M (2003). Rapid *in vitro* propagation of medicinally important *Ceropegia candelabrum*. Plant Cell Tiss Org Cult.

[10] Chavan JJ, Nalawade AS, Gaikwad NB, Gurav RV, Dixit GB, Yadav SR (2014). An efficient *in vitro* regeneration of *Ceropegia noorjahaniae*: an endemic and critically endangered medicinal herb of the Western Ghats. Physiol Mol Biol Plants.

[11] Chavan JJ, Nimbalkar MS, Gaikwad NB, Dixit GB, Yadav SR (2011). *In vitro* propagation of *Ceropegia spiralis* Wight - an endemic and rare potential ornamental plant of peninsular India. Proc Nat Acad Sci India Sect B.

[12] Chavan JJ, Gaikwad NB, Yadav SR (2013). High multiplication frequency and genetic stability analysis of *Ceropegia panchganiensis*, a threatened ornamental plant of Western Ghats: conservation implications. Sci Horti.

[13] Dhir R, Shekhawat GS (2014). Ecorehabilitation and biochemical studies of *Ceropegia bulbosa* Roxb: a threatened medicinal succulent. Acta Physiol Plantarum.

[14] Bhattacharyya P, Kumaria S, Tandon P (2016). High frequency regeneration protocol for *Dendrobium nobile*: a model tissue culture approach for propagation of medicinally important orchid species. South Afr J Bot.

[15] Murthy KSR, Kondamudi R (2011). Rapid shoot regeneration from thin cell layer explants of an endangered medicinal asclepiad *Ceropegia spiralis* L. Plant Tiss Cult Biotechnol.

[16] Aggarwal D, Kumar A, Sharma J, Reddy MS (2012). Factors effecting micropropagation and acclimatization of an elite clone of *Eucalyptus tereticornis*. In Vitro Cellular and Developmental Biology-Plant.

[17] Chandore AN, Nimbalkar MS, Gurav RV, Bapat VA, Yadav SR (2010). A protocol for multiplication and restoration of *Ceropegia fantastica* Sedgw: a critically endangered plant species. Curr Sci.

[18] Yadav SR, Chandore AN, Nimbalkar MS, Gurav RV (2009). Reintroduction of *Hubbardia heptaneuron* Bor, a critically endangered endemic grass in Western Ghats. Curr Sci.

